# Mental Health Problems among the Survivors in the Hard-Hit Areas of the Yushu Earthquake

**DOI:** 10.1371/journal.pone.0046449

**Published:** 2012-10-08

**Authors:** Zhen Zhang, Wenzhong Wang, Zhanbiao Shi, Li Wang, Jianxin Zhang

**Affiliations:** Key Laboratory of Mental Health, Institute of Psychology, Chinese Academy of Sciences, Beijing, People's Republic of China; The University of Hong Kong, Hong Kong

## Abstract

**Background:**

On April 14, 2010, an earthquake registering 7.1 on the Richter scale shook Qinghai Province in southwest China. The earthquake caused numerous casualties and much damage. The epicenter, Yushu County, suffered the most severe damage. As a part of the psychological relief work, the present study evaluated the mental health statuses of the people affected and identified the mental disorder risk factors related to earthquakes.

**Methods:**

Five hundred and five earthquake survivors living in Yushu County were investigated 3–4 months after the earthquake. Participant demographic data including gender, age, marital status, ethnicity, educational level, and religious beliefs were collected. The Earthquake-Specific Trauma Exposure Indicators assessed the intensity of exposure to trauma during the earthquake. The PTSD Checklist-Civilian version (PCL-C) and the Hopkins Symptoms Checklist-25 (HSCL-25) assessed the symptoms and prevalence rates of probable Posttraumatic Stress Disorder (PTSD) as well as anxiety and depression, respectively. The Perceived Social Support Scale (PSSS) evaluated subjective social support.

**Results:**

The prevalence rates of probable PTSD, anxiety, and depression were 33.7%, 43.8% and 38.6%, respectively. Approximately one fifth of participants suffered from all three conditions. Individuals who were female, felt initial fear during the earthquake, and had less social support were the most likely to have poor mental health.

**Conclusions:**

The present study revealed that there are serious mental problems among the hard–hit survivors of the Yushu earthquake. Survivors at high risk for mental disorders should be specifically considered. The present study provides useful information for rebuilding and relief work.

## Introduction

Earthquakes have been among the most destructive natural disasters throughout human history. On April 14, 2010, an earthquake registering 7.1 on the Richter scale (with a maximum intensity of 8) shook Qinghai Province in southwest China. The epicenter was located at Yushu County. This earthquake was the most destructive natural disaster in China since the 2008 Wenchuan earthquake in recent years. According to official toll, 2,698 people were killed, including 199 students, 270 more were listed as missing, 12,135 were injured, and approximately 200,000 were affected. This earthquake caused a 1.75-meter horizontal displacement and 0.60-meter vertical fault displacement in epicenter area. Some towns and villages were so badly destroyed, for example, at least 85% of houses collapsed in Yushu County seat, that many local inhabitants were settled in temporary tent communities (see http://news.sina.com.cn/c/2008-09-25/183514499939s.shtml for the final official statistics).

In addition to deaths, physical injuries and economic losses, strong earthquakes often result in serious mental health outcomes [1]–[4]. Although China frequently experiences earthquakes and other disasters, it is only in the last decade that the psychological aftermath of earthquakes has received attention. Wang et al. (2000) conducted a longitudinal survey on the onset and development of DSM-IV Posttraumatic Stress Disorder (PTSD) after the Zhangbei earthquake in North China and reported an onset rate of 18.8% within a 3-month period and an onset rate of 24.2% within a 9-month period [4]. The study also indicated that the rate of onset of PTSD within 9 months in severely affected village (30.3%) was higher than that in lightly affected villages (19.8%). The 2008 Wenchuan earthquake sparked a series of empirical studies on trauma-related mental disorders [5]–[10]. Three months after the Wenchuan earthquake, a survey revealed that the prevalence rates of probable PTSD in two communities differently affected by the earthquake were 37.8% and 13.0%, respectively [7]. Another survey two and a half months following the earthquake found that the prevalence rates of suspected PTSD in heavily and moderately damaged counties reached 45.5% and 9.4%, respectively [5]. Zhang and Ho (2011) found that PTSD symptoms affected 82.6% of survivors who lived in one of the most affected cities one to two months after the earthquake [9]. Wang et al. (2008) found that the rate of depression reached 31% among survivors living in a hard-hit town of the Wenchuan earthquake in the initial stage [10]. Liu et al. (2011) conducted a follow-up survey among children at two time-points (6 months and 12 months) after the Wenchuan earthquake, respectively. Results indicated that at two time-points the prevalence rates were respectively 23.3% and 22.7% for anxiety, 14.5% and 16.1% for depression, and 11.2% and 13.4% for PTSD [6].

To summarize the above studies, prevalence rates of mental disorders among the hard-hit survivors were far higher than those of lightly hit survivors. Second, the first few months (i.e., 0–6 months afterward) is often a high onset stage for mental disorders. Moreover, despite PTSD, anxiety and depression being identified as the most common mental problems [1], [3], [11], [12], [13], more studies focus on Acute Stress Disorder (ASD) or PTSD [2], [4], [14]–[19] only, and relatively fewer studies have examined the three mental symptoms concurrently [3], [6], [20].

Although the central and local governments devoted a large amount of manpower, materials and capital to the disaster relief work, little is known about the current mental health status of the Yushu earthquake survivors. Understanding mental problems and their associated risk factors is essential to identify vulnerable populations as well as implement effective assistance and post-disaster community reconstruction. To our knowledge, only few empirical studies had been published on psychological outcome of the Yushu earthquake. Yu et al. (2011) found that the rate of PTSD, which was assessed by the Impact of Events Scale-Revised, reached 73% among middle-school students two months after the Yushu earthquake [21]. Xu et al investigate the differences in acute stress reaction (ASR) between the Tibetan and Han people after the Yushu earthquake [22]. Results indicated that injured Tibetan people had lower scores of ASR symptoms than injured Han people.

The epicenter of the earthquake was Yushu County, located in the Qinghai-Tibet Plateau which is characterized by high altitudes, cold climate, and thin air. Yushu's dominant ethnic group is Tibetan, who constitute over 90% of the local population. The majority of Tibetan residents devoutly practice Buddhism, and influences of Buddhism permeate many aspects of their society and daily life. We do not know yet whether this unique culture, especially Buddhist belief, results in different mental health outcomes after the disaster. Griensven et al. (2006) have found a protective effect from Buddhist belief on anxiety and depression by investigating the survivors of Southeast Asia tsunami [3]. They believed that Buddhists tended to accept and overcome negative events after disaster and accident, and often adopt more positive life attitude in coping disasters [3]. In another study following L'Aquila earthquake in Italy indicated that religiosity helped to buffer against psychological distress caused by the earthquake [23]. Despite religion or spirituality often being perceived as a positive factor for those experiencing extremely difficult and negative life events, empirical findings on the role of religion in coping with trauma remain relatively scarce and uncertain [24].

Based on the above reasons and the absence of data regarding post-disaster psychology in this region, we conducted the present survey using adult survivors who live in the most severe affected regions 3–4 months after the Yushu earthquake. This survey is also a part of the post-earthquake relief work of the Institute of Psychology, Chinese Academy of Sciences (http://www.psych.cas.cn/xwzx/xlyz/). The primary aims of this study are to (1) assess the prevalence of probable PTSD, anxiety and depression, and (2) identify the risk and protective factors related to these mental disorders. Many studies recognize major risk and protective factors such as the intensity of exposure to trauma (e.g., loss of loved ones, loss of property, and injury) [2], [13], demographic characteristics (e.g., gender, age, marital status, and education level), and social support [1], [7]. The present study also will examine whether religious belief, mainly Buddhism, plays a part in buffering psychological symptoms.

Based on main findings of previous studies, the present study hypothesized: (1) that demographic characteristics, specifically female gender, older age, low education level, and unmarried status (including being single, divorced, separated, and widowed) are risk factors of psychological disorders; (2) religious beliefs (mainly Buddhism) may exert a positive role effect on mental health of survivors; (3) the extent and intensity of trauma exposure, specifically, bereavement, injury to self or family, witness of death, damage of property, houses' collapse, loss of livelihood and initial feelings of fear as common consequences of devastating disasters, are risk factors for poor mental health among survivors; and (4) social support is an important protective factor for mental health after disasters.

## Methods

### Procedures

Ethics approval for this study was obtained from the Ethics Committees of the Institute of Psychology, Chinese Academy of Sciences. The investigation was conducted anonymously. Verbal informed consent was obtained from each participant prior to interviewing. Written informed consent was not collected individually based on two considerations. First, as a part of the psychological relief program, this investigation was expected to minimize as much as possible the disturbance to survivors who have just experienced earthquake trauma. According to volunteers and social workers, requiring local survivors to sign their name without adequate explanations is very difficult, and might increase their worry for participating in the investigation. Second, according to observations of investigators, relative to oral commitments, local participants might interpret written informed consent as distrust. A statement approving this investigating procedure and verbal consent was signed between investigators and local community cadres. The Ethics Committees approved this consent procedure.

We conducted a cross-sectional survey 3–4 months after the earthquake. Given that most dwellings collapsed or were badly damaged, nearly all sampled residents lived in temporary tent communities. Psychological experts, volunteers, Tibetan undergraduates and local teachers comprised two teams of trained investigators.

The investigation was conducted using a door-to-door method. Sampling procedures of previous studies were followed [3], [8]: households were considered the basic sample unit, and only one member per household was chosen to represent that unit; participants were at least 16 years old and must have directly experienced the earthquake; the family member whose birthday was closest to the date of investigation was first selected as the participant. If this individual was unavailable for the survey, the household member with second closest birthday to the date of investigation was selected, this procedure continued until one participant was identified. Individuals who suffered from mental retardation or major psychoses (e.g., schizophrenia, major depressive disorder, and organic mental disorders) were excluded from the sample. Despite the fact that most participants are fluent in both Tibetan and Chinese languages, some (especially elderly residents and those with low levels of education) had difficulty understanding the Chinese versions of the questionnaires. Thus, two complementary methods were used to choose qualified participants: (1) Tibetan undergraduates and local teachers were employed on each team to help explain the questionnaire when needed; (2) When the closest birthday technique could not choose a qualified participant from a household, family nomination was instead used as an alternative sampling method. A family member who could fully understand the survey content and the procedure was then chosen as the participant from such households.

Demographic data included gender, age, marital status, ethnicity, education level, and religious beliefs. Religious believers were identified by asking participants to response to three options: A. no religious belief; B. believing in Buddhism; C. believing in other religions (please fill out in bracket).

### Participants

Participants mainly were sampled from several tent communities that temporarily settled survivors from different towns, a majority of whose residents came from Jiegu town. Jiegu suffered far more serious damage than other affected regions. Although it was reported that this earthquake affected over 200,000 people across 7 counties, the overwhelming majority of casualties and damage occurred in Jiegu.

A total of 505 residents were investigated (men = 270, women = 235). Participant age ranged from 16 to 87 years, and the mean age was 32.6 years (SD = 12.4). The proportions of Tibetan and Han peoples were 78.1% (n = 393) and 18.7% (n = 94) of the sample, respectively. All other minorities comprised less than 3.2% (n = 18). These demographics basically reflect the ethnic composition of towns in Yushu County. According to official data, Tibetan account for about 79% of the total population of Jiegu town (about 23,000 people), Han and other minority nationalities account for about 20% (see: http://www.qh.xinhuanet.com/yushu/ysjj.htm). We only compared Tibetan with Han people for the latter analyses. Table 1 shows the participant demographic information.

**Table 1:** Participant demographic information (n = 505).

	n	%
Gender		
Men	270	53.5
Women	235	46.5
Age		
19 and younger	45	8.9
20–29 years	202	40.3
30–39 years	115	22.8
40–49 years	91	18.1
50 and older	51	9.9
Ethnicity		
Tibetan	393	78.1
Han	94	18.7
Other minorities	18	3.2
Religious Beliefs		
No	80	15.8
Yes	420	83.2
Missing	5	1.0
Marital Status		
Unmarried	213	42.2
Married	275	54.5
Missing	17	3.3
Education Level		
Elementary or lower	171	33.9
Middle school	170	33.7
College or higher	144	28.5
Missing	20	3.9

### Measures

#### Earthquake-Specific Trauma Exposure Indicators

Referring to recent studies on Wenchuan earthquake [7]–[8], eight items were developed to evaluate the extent of exposure to traumatic events during the Yushu earthquake: (1) whether the participant had one or more family members who died or went missing from the earthquake; (2) whether the participants witnessed the death of someone; (3) whether the participant had been physically injured by the earthquake; (4) whether the participant had family members who were disabled by the earthquake; (5) whether the participant's housing was destroyed in the disaster; (6) the extent of damage of properties in the earthquake (rated on a five-point Likert scale *1 = lost no or little property, 2 = lost a small part of property, 3 = lost about half of property, 4 = lost most of property, and 5 = lost almost all property*); (7) the impact of the earthquake on their livelihood or work (rated on a five-point Likert scale *1 =  not at all, 2 = little, 3 = to some degree, 4 = strong, and 5 = extreme*); and (8) the initial intensity of fear when the earthquake occurred (rated on a four-point Likert scale *1 = not at all, 2 = to some degree, 3 = strong, 4 = extreme*). Participants answered “yes” or “no” to the first five items. The Cronbach alpha coefficient of first five items was 0.53.

#### PTSD Checklist-Civilian version (PCL-C)

The PCL-C is one of the most commonly used instruments to assess PTSD [25]–[26]. Its 17 items measure the three clusters of symptoms that map onto diagnostic Criteria B (re-experiencing), C (avoidance/numbing), and D (hyper-arousal) for PTSD in the DSM-IV. Participants indicated the extent to which each symptom was correlated with the earthquake on a scale from 1 (*not at all*) to 5 (*extremely*). The Chinese version of the PCL-C has sound reliability and validity [8], [27]–[28]. In the present study, a score of forty-four was used as a measure to screen probable PTSD. This score was demonstrated the optimally efficient cutoff score for clinical diagnosis of PTSD [27], [29]–[30]. In this study, the internal consistency of the total scale was 0.91, and the internal consistencies of the re-experiencing, avoidance/numbing, and hyper-arousal subscales were 0.82, 0.81, and 0.83, respectively.

#### The Hopkins Symptoms Checklist-25 (HSCL-25)

The HSCL-25 screens for symptoms of anxiety and depression [31]–[32] using 10- and 15-item subscales, respectively. Scores range from 1 *(not at all)* to 4 *(extremely)*. Symptoms were assessed with regard to the past month. A mean cumulative score of subscales greater than 2.00 indicates anxiety or depression [33]. The Chinese version of the HSCL-25 showed satisfactory reliability [8]. In this study, the internal consistencies for the anxiety and depression subscales were 0.90 and 0.90, respectively.

Note that due to the lack of clinical diagnoses, the mental disorder screenings via the above scales do not necessarily indicate psychopathologies; thus, this paper merely refers to probable PTSD, anxiety and depression.

#### Perceived Social Support Scale (PSSS)

The PSSS evaluates subjective social support from friends and family [34]. Numerous empirical studies demonstrate that social support has a protective function with regard to post-disaster mental health [7], [35]. The PSSS contains 12 items with scores ranging from 1*(strongly disagree)* to 7 *(strongly agree)*. The reliability and validity of the Chinese Version of the PSSS are satisfactory in Chinese sample [36]. The Cronbach alpha coefficient of the PSSS was 0.88 in this study.

### Data Analyses

Descriptive statistics were computed for demographic characteristics, trauma exposure indicators, social support measures, and the prevalence rates of probable PTSD, anxiety, and depression. Bivariate logistic regressions screened for the risk factors of probable PTSD, anxiety, and depression by exploring the roles of the above independent variables, consecutively. Multivariate logistic regression analyses identified the independent role of each risk factor for the mental symptoms. Based on public health studies [1], [3], all the variables that were statistically significant (p<.05) in the bivariate analyses were included in the multivariate model. All analyses were conducted using SPSS Version 15.0 for Windows.

## Results

Descriptive statistics and correlation analyses of the severity of PTSD, anxiety and depression symptoms are presented in Table 2. The results indicate that there are significant correlations among PTSD, anxiety and depression symptoms, which suggest that the occurrence of PTSD among earthquake survivors is concomitant with anxiety and depression. The prevalence rate of probable PTSD was 33.7% (n = 170) based on the PCL-C cut-off score of 44. The prevalence rates of probable anxiety and depression were 43.8% (n = 220) and 38.6% (n = 194), respectively, based on the HSCL-25 average scores of 2.00 (Table 3). Twenty point five percent of participants (n = 103) had all three mental problems.

**Table 2:** Descriptive statistics and correlations of the severity of PTSD, anxiety and depression symptoms.

	Mean(total)	SD(total)	Mean	SD	1	1.1	1.2	1.3	2	3
1 PTSD(17)	39.73	12.40	2.34	0.73	1.00					
1.1 Re-experiencing(5)	12.11	4.43	2.42	0.89	0.83**	1.00				
1.2 Avoiding/Numbing (7)	14.78	5.35	2.11	0.76	0.87**	0.56**	1.00			
1.3 Hyper-arousal(5)	12.84	4.66	2.57	0.93	0.87**	0.62**	0.64**	1.00		
2 Anxiety(10)	19.33	6.15	1.93	0.62	0.72**	0.55**	0.61**	0.69**	1.00	
3 Depression(15)	27.73	8.67	1.85	0.58	0.65**	0.41**	0.63**	0.62**	0.69**	1.00

* P<0.05,

** P<0.01; the parentheses include the number of items in each scale or subscale.

**Table 3:** Bivariate logistic regression analyses of the effects of demographics, trauma exposure and social support on the odds of probable PTSD, anxiety and depression.

	PTSD		Anxiety		Depression	
Variable	No. (%)	OR(95 CIs)	No. (%)	OR(95 CIs)	No.(%)	OR(95 CIs)
Overall	170 (33.7)		220 (43.8)		194 (38.6)	
Gender	504#		502#		503#	
Men	69 (25.7)	1	102 (38.1)	1	93 (34.6)	1
Women	101 (43.0)	2.18 (1.50, 3.18)**	118 (50.4)	1.66 (1.18, 2.36)**	101 (43.2)	1.44 (1.00, 2.06)*
Age	503#	1.03(1.01,1.04)**	504#	1.03(1.01,1.04)**	502#	1.02(1.01,1.04)**
Marital status	488#		485#		486#	
Married	67 (31.6)	1	126(46.0)	1	111(40.5)	1
Unmarried	98 (35.6)	1.20 (0.82, 1.75)	86(40.8)	0.81 (0.56,1.16)	77 (36.3)	1.20 (0.83,1.73)
Nationality	486#		484#		485#	
Han	18 (19.1)		40 (43.0)	1	35 (37.6)	1
Tibetan	146 (37.2)	2.51 (1.44, 4.36)**	173 (44.2)	1.05 (0.67, 1.66)	153 (39.0)	1.06(0.67, 1.69)
Education	485#		483#		484#	
College or higher	40(27.8)	1	48(33.3)	1	50 (34.7)	1
Middle school	45 (26.5)	0.94(0.57,1.54)	65(38.5)	1.25(0.79,1.99)	58(34.3)	0.98(0.62,1.57)
Elementary school or below	80 (46.8)	2.29 (1.43, 3.67)**	98(57.6)	2.72 (1.72, 4.32)**	80 (46.8)	1.65(1.05,2.61)*
Religious beliefs	499#		497#		491#	
No	11(15.9)	1	27(39.1)	1	161(37.4)	
Yes	158(37.0)	3.07(1.56,6.02)**	192(44.9)	1.27(0.75,2.13)	29(47.5)	1.52(0.88,2.21)
Bereavement	474#		472#		473#	
No	114(32.3)	1	154(43.9)	1	131(37.2)	1
Yes	52(43.0)	1.58 (1.03, 2.44)*	60(49.6)	1.26 (0.83, 1.90)	56(46.3)	1.45(0.91,2.21)
Witnessed death	500#		498#		499#	
No	16 (26.2)		21(34.4)	1	20(32.8)	1
Yes	153(34.9)	1.50(0.82, 2.75)	197(45.1)	1.56(0.89,2.74)	173(34.5)	1.34(0.76,2.36)
Injury of body	502#		500#		501#	
No	95(29.3)	1	124(38.4)	1	103 (31.9)	1
Yes	75(42.1)	1.75 (1.20, 2.57)**	94(53.1)	1.82(1.25,2.63)**	89 (50.0)	2.14 (1.47,3.11)**
Disability of family members	475#		473#		474#	
No	95(30.4)		135(43.3)	1	111(35.6)	
Yes	75(42.0)	1.66(1.12, 2.46)**	74(46.0)	1.11(0.76,1.63)	75(46.3)	1.56(1.06,2.30)*
Loss of house	502#		500#		501#	
No	12(21.1)	1	18 (31.6)	1	20(35.1)	1
Yes	156(35.1)	2.02(1.04, 3.94)*	200 (45.1)	1.78 (0.99, 3.22)	172(38.7)	1.17 (0.66,2.08)
Loss of property	503#	1.58(1.31, 1.89)**	501#	1.36(1.16,1.59)**	502#	1.45(1.23,1.72)**
Loss of livelihood	499#	1.53(1.22, 1.92)**	497#	1.16(0.94, 1.43)	498#	1.55(1.24,1.93)**
Initial fear	493#	1.61(1.30, 2.01)**	491#	1.96(1.58, 2.43)**	492#	1.12 (0.92, 1.36.)
Social supports	502#	0.98 (0.97, 0.99)**	499#	0.98 (0.97, 0.99)**	500#	0.97 (0.95, 0.98)**

OR = odds ratio; CIs = confidence intervals.

* P<0.05,

** P<0.01.

# Numbers within categories may not add up to 505 for some variables due to missing data.

The results of the bivariate analyses indicate that women (OR = 2.18, 95%CI = 1.50–3.18, p<.01), Tibetan people (OR = 2.51, 95%CI = 1.50–3.18, p<.01), participants with older age (OR = 1.03, 95%CI = 1.01–1.04, p<.01), elementary school or below education (OR = 2.29, 95%CI = 1.43–3.67, p<.01), who are religious believers (mainly Buddhist) (OR = 3.07, 95%CI = 1.56–6.02, p<.01) were more likely to report scores above the cut-off for probable PTSD compared with participants without these qualities. Persons with more serious exposures to earthquake trauma (excluding witnessing death), such as bereavement (OR = 1.58, 95%CI = 1.03–2.44, p<.05), injury of body (OR = 1.75, 95%CI = 1.20–2.57, p<.01)), disability of family members caused by the earthquake (OR = 1.66, 95%CI = 1.12–2.46, p<.01), loss of houses (OR = 2.02, 95%CI = 1.04–3.94, p<.05), loss of property (OR = 1.58, 95%CI = 1.31–1.89, p<.01) and loss of livelihood (OR = 1.53, 95%CI = 1.22–1.92, p<.01), were more likely to report scores above the cut-off for probable PTSD compared with participants without these qualities. The results of bivariate analyses also indicated that women (OR _anxiety_ = 1.66, 95%CI = 1.18–2.36; OR _depression_ = 1.44, 95%CI = 1.00–2.06), participants with older age (OR _anxiety_ = 1.03, 95%CI = 1.01–1.04; OR _depression_ = 1.02, 95%CI = 1.01–2.04), elementary school or below education (OR _anxiety_ = 2.72, 95%CI = 1.72–4.32; OR _depression_ = 1.65, 95%CI = 1.05–2.61), who were injured (OR _anxiety_ = 1.82, 95%CI = 1.25–2.63; OR _depression_ = 2.14, 95%CI = 1.47–3.11) and suffered more loss of property (OR _anxiety_ = 1.36, 95%CI = 1.16–1.59; OR _depression_ = 1.45, 95%CI = 1.23–1.72), were more likely to report scores above the cut-off for anxiety and depression. In addition, participants with stronger initial fears during the earthquake were more likely to report scores above the cut-off for probable anxiety (OR = 1.96, 95%CI = 1.58–2.43, p<.01). Individuals who suffered the loss of their livelihoods (OR = 1.55, 95%CI = 1.24–1.93, p<.01) and had family members with disability caused by the earthquake (OR = 1.56, 95%CI = 1.06–2.30, p<.05) were more likely to report scores above the cut-off for probable depression. Scores on the measure of social support demonstrated a protective effect for suffering probable PTSD (OR = 0.98, 95%CI = 0.97–0.99, p<.01), anxiety (OR = 0.98, 95%CI = 0.97–0.99, p<.01), and depression (OR = 0.97, 95%CI = 0.95–0.98, p<.01) (Table 3).

Although some demographic and exposure to earthquake trauma variables were related to psychological disorders in the bivariate analyses, the independent role of each variable should be examined after adjustment for confounding. Thus, full-adjusted multivariate analyses are conducted to identify the independent role of each variable for PTSD, anxiety and depression. The results of these analyses indicated that only female gender (OR _PTSD_ = 2.12, 95%CI = 1.37–3.37; OR _anxiety_ = 1.63, 95%CI = 1.08–2.44; OR _depression_ = 1.61, 95%CI = 1.07–2.43) and low PSSS scores (OR _PTSD_ = 0.98, 95%CI = 0.96–0.99; OR _anxiety_ = 0.98, 95%CI = 0.96–0.99; OR _depression_ = 0.97, 95%CI = 0.95–0.98) were associated with the presence of probable PTSD, anxiety and depression. Initial fear during the earthquake was significantly associated with probable PTSD (OR = 1.38, 95%CI = 1.10–1.87) and anxiety (OR = 1.86, 95%CI = 1.48–2.36). In addition, low levels of education were significantly associated with probable anxiety (OR = 1.78, 95%CI = 1.03–3.10), and injuries were significantly associated with probable depression (OR = 1.72, 95%CI = 1.10–2.67) (Table 4).

**Table 4:** Multivariate logistic regression analyses of the factors significantly associated with PTSD, anxiety and depression.

	PTSD		Anxiety		Depression	
Variable	OR(95 CI)	P	OR(95 CI)	P	OR(95 CI)	P
Gender						
Men	1		1		1	
Women	2.15 (1.37, 3.37)	0.001	1.63(1.08, 2.44)	0.020	1.61(1.07,2.43)	0.023
Age	1.02(0.99, 1.04)	0.093	1.01(0.99, 1.03)	0.127	1.01(0.99, 1.03)	0.171
Nationality						
Han	1					
Tibetan	1.06 (0.43, 2.60)	0.898				
Education						
College or higher	1		1		1	
Middle school	1.05(0.57, 1.94)	0.875	1.31(0.78,2.19)	0.306	1.10(0.65,1.85)	0.717
Elementary school or lower	1.21(0.67, 2.21)	0.528	1.78(1.03,3.10)	0.040	1.10(0.64,1.90)	0.722
Religious beliefs						
No	1					
Yes	2.54 (0.86, 7.52)	0.093				
Bereavement						
No	1					
Yes	1.20(0.71,2.01)	0.500				
Bodily injury						
No	1		1		1	
Yes	1.22 (0.74,1.99)	0.437	1.27(0.82, 1.95)	0.286	1.72(1.10, 2.67)	0.017
Disability of family members						
No	1				1	
Yes	1.22(0.72,2.07)	0.456			1.02(0.65,1.61)	0.925
Loss of home						
No	1					
Yes	1.46 (0.59,3.59)	0.412				
Loss of property	1.14(0.87,1.48)	0.351	1.18(0.97,1.42)	0.100	1.15(0.92,1.43)	0.228
Loss of livelihood	1.29(0.94,1.78)	0.113			1.27(0.96,1.69)	0.098
Initial fear	1.38 (1.10,1.87)	0.008	1.86 (1.48, 2.35)	0.000		
Social support	0.98(0.96,0.99)	0.024	0.98(0.96,0.99)	0.003	0.97 (0.95,0.98)	0.000

All significant univariate logistic analysis variables (i.e., P-values equal to 0.05 or less) were included in the multivariate logistic regression.

## Discussion

The present study revealed significant levels and prevalence of psychological problems among the survivors of the Yushu earthquake. Consistent with previous studies on mental problems among extremely affected survivors in the initial stages of an earthquake [5], [7], the present study found that approximately one third of participants suffered from probable PTSD (33.7%). The prevalence rates of probable anxiety (43.8%) and depression (38.6%) were even higher. According to several national epidemiological surveys, the population prevalence rates of these disorders in comparable regions of China not affected by recent natural disasters ranged from 1% to 10%, and were far below those of current survey [37]–[38]. Approximately one fifth of participants suffered from all three mental conditions. This finding means that the symptoms of anxiety and depression are common in the heavily affected region. The prevalence rates of PTSD were roughly comparable to outcomes of earthquake-related studies that were conducted in first a few months after earthquake among the severe affected survivors [3], [5], [7], [9]–[10]. It should be pointed out, however, that the diversities in many aspects in earthquake-related studies (such as destructive extent, affected population, assessment methodologies, instruments, and timing of investigation) made it difficult to conduct an enough comparison on morbidity and risk factors of mental disorders across earthquake disasters [2].

Such high prevalence rates are not surprising considering that this earthquake was one of the most destructive in this area in the past several decades. Qinghai province to which Yushu County belongs lies on the Qinghai-Tibet Plateau of western China, within what is called the “life forbidding zone”, and is also a relatively undeveloped province of China. Historically, Yushu is among the most seismically active areas in China. Owing to sparse and scattered population (less than two people/km^2^), earthquakes often result in few casualties and economic losses. Unfortunately, the epicenter of this strong earthquake was near populous Jiegu town, the Yushu County seat. In addition, most of the adobe dwellings where residents live were easily destroyed which aggravated losses and increased causalities. Thus, the psychological shock that this disaster brought is undoubtedly immense for the residents who lived largely uneventful lives.

Immediately following the Yushu earthquake, central and local government and nongovernmental organizations adopted various emergency measures to cope with this disaster, including evacuating and settling survivors, transferring the wounded and students to other cities or regions, and continuously sending relief supplies and rescuers into the hard-hit areas. Although extremely adverse natural environment (such as cold climate, thin air, and high altitude) and economic conditions (such as lack of transport facilities) brought huge pressure and challenge to disaster relief and rehabilitation work, above effective measures to a certain degree relieved post-earthquake fear and anxiety of survivors.

The findings identified several related risk for three mental disorders. Among the demographics, gender differences in the prevalence rates were the most robust. Compared with men, women were approximately twice as likely to develop PTSD and 60% more likely to report anxiety and depression when exposed to earthquake trauma. This finding agrees with the conclusions of many studies of the gender differences in disaster psychology [4], [15], [39]–[40]. Kumar et al. (2007) reported that women were more than twice as likely as men to develop PTSD two months after the December 2004 tsunami [17]. Some studies indicated that women were more sensitive to threats, were less likely to use effective coping strategies, and tended to interpret disasters more negatively [40]–[43]. Lower self-efficacies, higher peritraumatic emotions, subordinate familial roles and limited social-economic resources compared with men, may also contribute to the reported greater vulnerability among women to terror and disasters [40]–[43]. In addition, it is possible that women are more sensitive to negative events and more tend to express emotion. When faced with this unexpected disaster, men were probably just as frightened as women, but in this retrospective study, they may be significantly under-reporting their fear and grief, but generally women tend to be more willing to admit to their negative emotions. Additional studies are needed to clarify which of these explanations for the women's apparent vulnerability is likely.

Note that some of the variables that predicted mental problems from earthquake-specific trauma exposure in bivariate analysis (e.g., bereavement and economic losses) were not significant in the multivariate analyses. These results are inconsistent with the conclusions of many empirical studies examining the extent of disaster exposure; specifically, high-level losses are one of the most important risk factors for developing mental disorders [2], [20], [44]. Nevertheless, these findings do not mean that bereavement and economic losses are of no importance. First, death, missing people and large economic losses were commonplace in this severely affected region. This investigation found that approximately one fourth of survivors experienced the death of one or more family members (note that missing people are often declared dead one year after earthquakes), 87 percent of participants witnessed death during the earthquake, and approximately 89 percent of participants lost their homes. Second, the findings of some studies suggest that the effect of trauma exposure becomes more prominent over time [4], [7]. In the initial aftermath, the most important task is rebuilding one's house and collecting relief materials to meet basic survival needs. People are then able to resume work and collect themselves. However, the grief over losing one's home and the bereavement regarding other losses maybe becomes more prominent as time passes. Thus, longitudinal studies are necessary to examine the long-term impacts of earthquake-specific trauma exposure on mental health.

The results of the present study indicated that the intensity of initial fear predicted positive symptoms of PTSD and anxiety. Consistent with the results of recent studies [7]–[8], [11], the initial feeling of fear was a fairly robust predictor of psychological disorders. The intensity of this initial fear represents one's personal experience to a disaster and is included as PTSD Criterion A2 in the DSM-IV (1994). First, the intensity of initial fear is linked to trauma exposure in general. People with the strongest fears have often suffered the severest traumas. Second, the intensity of initial fear may signify susceptibility of specific personality to tragedy and disaster. Individuals high in neuroticism are more reactive to adverse events; thus, they may be more likely to develop mental disorders [45]–[46].

The results also indicated that social support has a significant positive effect on all three mental problems. Consistent with previous studies on posttraumatic psychological health, the present study confirmed that social support has a protective function [1], [7], [47]–[48]. Cook and Bickman (1990) found that social support and mental health symptomatology were significantly correlated during the intermediate period following a flood [48]. Acierno and his colleagues (2007) found that high social support preceding hurricanes protected against generalized anxiety disorder, depressive disorder and PTSD [1]. When facing forces of nature such as earthquakes, social relationships, materials and spiritual encouragement from families and friends positively offset the negative effects of trauma.

The present study found no protective role of religion belief in buffering against psychological trauma. Despite bivariate analysis using logistic regression indicating that believers were approximately three times as likely to develop PTSD compared with non-believers. However, multivariate analysis controlling the confounding effect of other variables indicated that independent role of religion belief in PTSD is insignificant. Although a few studies demonstrated a positive effect of religion on mental health after disaster [3], [23], the conclusion remains indeterminate on relationship between religion and mental health. A review of empirical studies on the relationship between religion and traumatic stress has produced mixed findings [24]. Wang and Han (2009) found that aging Buddhism believers showed a worse health status in the aftermath of Wenchuan earthquake [49]. However, it may be premature to conclude that religion is inconsequential to mental health or mental disorders. First, when confronted with an unexpected catastrophe, Buddhism may exert both positive and negative influence in psychological response [23]. Thus, a believer' religious coping consists of both positive and negative coping, which are expected to yield results in the opposite directions. Second, methodology factors might have contributed to current result. To our knowledge, the majority of locals believe in Tibetan Buddhism, and religious influences permeate many aspects of their society and daily life. For a society which is predominantly Buddhist of greater significance may be not to identify believer or nonbeliever, but rather to explore one's strength of religious faith and specific religious practices. Investigators really are faced with some difficulties to achieve this in the early stage when a lot of time and effort were invested in rebuilding works. In addition, the present study used a cross-sectional design to collect data, so we can't establish direction of causation. That is, we can't rule out the possibility that some residents became religious or stopped being religious after experiencing the earthquake trauma (for example, some non-believers began to worship Buddha or God because they survived the disaster; and some believers might not longer believe any god owing to death of their loved one). Falsetti et al. (2003) found PTSD group was more likely to report changes in religious beliefs following the first/only traumatic event, generally becoming less religious [50]. Thus, future in-depth interviews and longitudinal studies might contribute to a better understanding the role of beliefs in coping with disasters and traumas, and help to conduct psychological relief work integrating with local cultural characters.

The present study has several limitations that should be mentioned. First, although trained investigators conducted our survey adequately, this survey was essentially a self-report assessment. There were no clinical diagnoses of functional impairments or PTSD, anxiety and depression symptoms, and the PCL-C and HSCL do not necessarily indicate the presence of psychopathology. Thus, this study may overestimate the prevalence of mental illness relative to a clinical diagnosis. Second, the study sampled a decimated county, thus, the conclusions should not be generalized to lightly affected regions. But, compared with Wenchuan earthquake, severely affected areas and populations by the Yushu earthquake are comparatively centralized. Third, this survey was performed in a minority district with unique religious culture. However, only one self-report item was used to identify the believer and nonbeliever. In this region where Buddhism is so prevalent among local residents, more detail questionnaires and deep interview may be necessary to explore the relationship among strength of religious faith, style of coping with disasters, and various symptoms of psychological disorders. Fourth, despite our door-to-door method, the sample distribution did not completely correspond to the population distribution in this region; specifically, our sample was composed of participants with higher education levels than average. According to statistics bulletin of National Bureau of Statistics of China, the population who have received higher education account for 8.6% of total population of Qinghai, which was obviously lower than ratio of the higher education population of current sample (28.5%) (http://www.stats.gov.cn/tjgb/rkpcgb/). This distribution was partly because family members with more education were often nominated as participants. These participants more easily understood the content and the procedure of the investigation. This sample bias partly discounts the generality of our conclusions.

Despite these limitations, to our knowledge, this study was among the first to investigate the mental health of the Yushu earthquake survivors. Furthermore, it contributed new knowledge regarding the psychological aftermath of catastrophic natural disasters in China, especially in Tibetan-inhabited area. Rather than the mere examination of PTSD prevalence found in most disaster studies, the present study also found that anxiety and depression are common mental health problems in the early stages after the earthquake. From an intervention and health-enhancement perspective, the present study also provides useful information for rebuilding and relief work. People at high risk for mental disorders, especially women and those with fewer social relationships and less social support, should be specifically considered. Our findings suggest that recovering and improving mental health is rarely just about psychological service; in addition, mental health should be related to rebuilding homes, communities and social support systems. Given the extreme lack of psychology research on this cultural environment, building adequate psychological service sites and training local people for psychological relief work is needed immediately.
